# The Bergen 4-Day Treatment (B4DT) for Obsessive-Compulsive Disorder: Outcomes for Patients Treated After Initial Waiting List or Self-Help Intervention

**DOI:** 10.3389/fpsyg.2020.00982

**Published:** 2020-05-27

**Authors:** Gunvor Launes, Kristen Hagen, Lars-Göran Öst, Stian Solem, Bjarne Hansen, Gerd Kvale

**Affiliations:** ^1^Sørlandet Sykehus, Kristiansand, Norway; ^2^Department of Clinical Psychology, University of Bergen, Bergen, Norway; ^3^Bergen Center for Brain Plasticity, Haukeland University Hospital, Bergen, Norway; ^4^Møre and Romsdal Hospital Trust, Molde, Norway; ^5^Department of Psychology, Stockholm University, Stockholm, Sweden; ^6^Department of Psychology, Norwegian University of Science and Technology, Trondheim, Norway; ^7^Center for Crisis Psychology, Faculty of Psychology, University of Bergen, Bergen, Norway

**Keywords:** obsessive-compulsive disorder, ERP, RCT, B4DT, self-help, waiting list, follow-up

## Abstract

The Bergen 4-day treatment (B4DT) for obsessive-compulsive disorder (OCD) was recently tested in a randomized controlled trial, where the results showed that the B4DT was more effective than a self-help intervention (SH) and a waiting list condition (WL). Patients in the SH and WL condition still in need of treatment after the first intervention (*N* = 26; 13 from each condition) were offered the B4DT. None of the patients declined participation, and there were no dropouts. At post-treatment 59.5% were in remission, 31.0% had treatment response, and 9.5% showed no change. At 3-month follow-up 71.4% were in remission, 19.0% had treatment response, and 9.5% showed no change. There were also significant improvement in self-reported symptoms of OCD, generalized anxiety symptoms, and depressive symptoms. The results are in line with what we previously have found for the B4DT in a number of effectiveness studies. In addition, the results indicate that the patients who previously had received an unsuccessful SH intervention and patients who had first been in a WL condition, profited as much as patients who had received the B4DT as the initial intervention. Implications for clinical guidelines and for dissemination of the B4DT are discussed.

## Introduction

Obsessive-compulsive disorder (OCD) is a disabling disorder characterized by recurrent, unwanted thoughts, images, or urges (obsessions) and/or repetitive behaviors or thoughts (compulsions) (American Psychiatric and Association, 2013). Cognitive behavioral therapy (CBT) with exposure and response prevention (ERP) is a well-established treatment for patients with OCD (Rosa-Alcázar et al., 2008; Öst et al., 2015). Meta-analyses (e.g., Öst et al., 2015) of randomized controlled trial (RCT) suggest that approximately 65% of patients achieve a reliable treatment response and 50% are in remission following treatment. Treatment can be given individually and in groups and with different intensity and the results are quite similar (Rosa-Alcázar et al., 2008; Olatunji et al., 2013; Öst et al., 2015). Several studies suggest that intensive (in- and outpatient) treatment could be effective in treating adults with OCD (Jónsson et al., 2015; Veale et al., 2016), but also for children with anxiety (Öst and Ollendick, 2017). These treatments are, however, often not available for the patients (Shafran et al., 2009). The National Institute for Health and Care Excellence (NICE, 2005) thus recommends a stepped care model where the first intervention is “brief individual CBT (including ERP) using structured self-help materials” since these approaches can be easily disseminated. Self-help interventions are documented effective, although with smaller effect sizes than face-to-face treatment (Pearcy et al., 2016).

Since 2012, the OCD-team in Bergen, Norway, has developed a highly concentrated exposure based treatment format for patients with OCD, the Bergen 4-day treatment (B4DT). During four consecutive days, OCD patients receive individually tailored and therapist assisted exposure treatment delivered in a group setting, where the patient–therapist ratio is 1:1. The approach is highly attractive to the patients, and there is basically no attrition. A number of open trials indicate that more than 90% of the patients can expect to respond to treatment with nearly 70% being recovered 1–4 years after participating in the B4DT (Hansen et al., 2018, 2019). The B4DT has also been piloted outside the Bergen group (Kvale et al., 2018; Launes et al., 2019a, b), and in a different country (Davíðsdóttir et al., 2019), with the same results.

Recently, the first randomized controlled trial (RCT) of the B4DT was completed (Launes et al., 2019b), where it was compared to a self-help (SH) intervention as well as a waiting list (WL) condition. Results showed that 93.8% responded to treatment in the B4DT condition. For patients in the SH condition, 12.5% responded, while none of the patients on WL had a treatment response. At 3-month follow-up, 69% were remitted and 31% were improved. At 6-month after treatment, 81% were in remission, 6% were improved, and 13% unchanged. Thus, the first controlled trial of the B4DT supported the positive findings from the open trials.

The patients who were in need of further treatment after the SH or WL condition were offered the B4DT, which implies that for the patients who first received the SH, the National Institute for Health and Care Excellence (NICE, 2005) guidelines of a stepped care approach were followed. Clearly, the NICE recommendations assume that patients who have first received unsuccessful SH are not at a disadvantage when they proceed to a therapist-assisted intervention, but the documentation for this is sparse. Thus, in the current article we will investigate the following questions: (1) Are clinical changes after the B4DT also seen in the patients with prior experience of SH or a WL, and are the changes maintained at 3-month follow-up? (2) Do the patients who had received an unsuccessful SH treatment profit as much as patients who had received the B4DT as the initial intervention?

## Methods

### Participants and Design

From 2016 to 2017 a randomized controlled trial (RCT) was conducted at Solvang Community Psychiatric Service (Sørlandet Hospital) in Norway (Launes et al., 2019b). The RCT compared the effects of B4DT to the effects of a SH intervention and a WL. Included participants consisted of consecutive referrals to a specialized OCD-team at the hospital. Patients were referred from their general practitioner or their local outpatient clinic. Referrals were based upon a suspicion of OCD.

**FIGURE 1:**
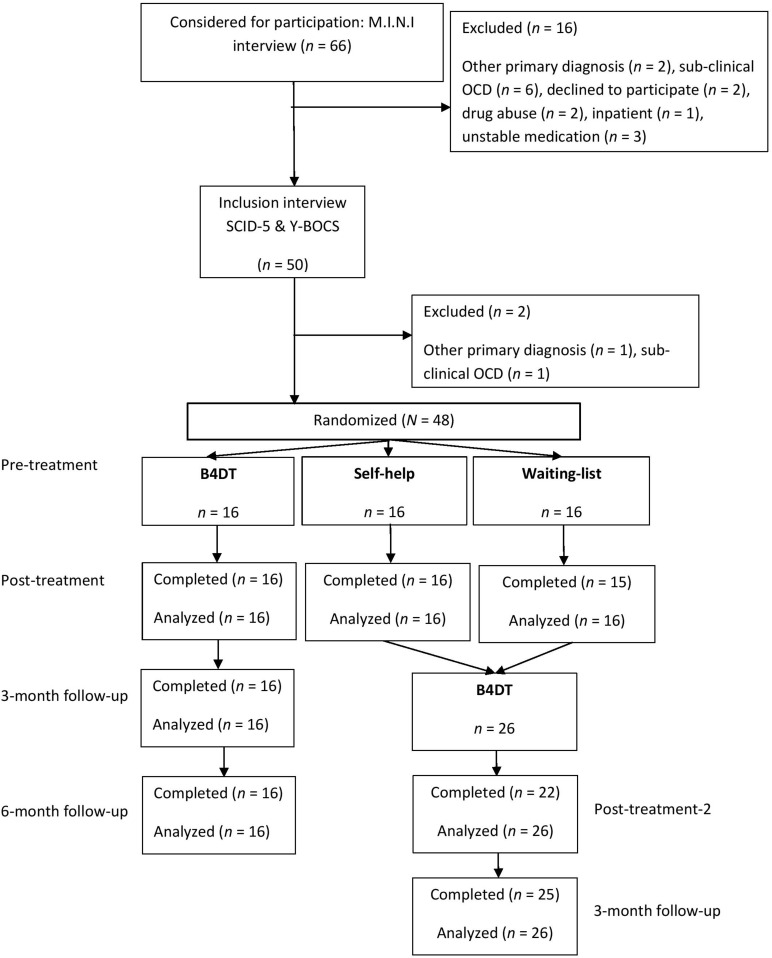
Flow chart.

The inclusion criteria for the study were: (a) signed informed consent, (b) ≥18 years, (c) met DSM-5 criteria for OCD, (d) Yale-Brown Obsessive Compulsive Scale (Y-BOCS) score ≥16, and (e) spoke Norwegian fluently. The exclusion criteria for the study were: (a) meeting DSM-5 criteria for bipolar disorder, psychosis, or ongoing substance abuse/dependence, (b) intellectual disability based on previous medical history, (c) an eating disorder in need of medical attention, (d) not willing to refrain from anxiolytics during treatment, (e) ongoing suicide ideation, (f) SSRI-treatment initiated or dosage changed during the last four weeks before starting treatment, and (g) living far away from the treatment center (>1.5 h). Patients with prior CBT for OCD were not included due to a nationwide trial targeting this population. The patients who were not helped after a SH condition or a WL were offered the B4DT (Launes et al., 2019b). A flow-chart of the study is presented in Figure 1.

Two of the patients who had received the SH intervention had improved according to their own evaluations and did not want further treatment and one patient who had received SH moved to another part of the country. Thus, 13 patients in the previous SH condition started treatment with the B4DT. Of the 15 patients who previously completed the WL condition, one moved to another part of the country, and another received inpatient treatment due to pregnancy complications, leaving 13 patients who started the B4DT. All patients who had received B4DT as the immediate intervention (*n* = 16) function as a comparison condition at follow-up. The 26 patients who had received either WL or SH condition were assigned to the first available B4DT group.

#### Background Data

Table 1 displays a summary of the participants’ demographic data, diagnostic information and pharmacological treatment. There was no difference in pre-treatment Y-BOCS scores between patients who had previously been in the SH or the WL condition, *F*(1,24) = 0.121, *p* = 0.731. In addition, there were no significant differences between these two groups on any other pre-treatment variable. All patients but one were classified as having severe OCD (Y-BOCS score of 24 or more) at pre-treatment.

**TABLE 1:** Background characteristics of the combined waiting list- and self-help condition (*N* = 26).

	***M* (*SD*)**
Age	28.08(6.69)
Age of OCD onset	13.19(7.44)
Number of comorbid disorders	2.65(1.50)

	*N*(%)

Females	21(80.8)
Single	13(50.0)
Student	5(19.2)
Employed	9(34.6)
On social benefits	12(46.2)
OCD in the family	8(30.8)
Psychotropic medication	11(42.3)
Any comorbid disorder	24(92.3)
Comorbid depression	14(53.8)
Comorbid anxiety	16(61.5)
Other comorbidity	10(38.5)

*Three patients had five comorbid disorders, six had four, four had three, seven had two, and four patients had one comorbid disorder. The “other comorbidity” category included bulimia, insomnia, ADHD, and skin picking. There were no significant differences between the SH and WL group on these variables, except for “other comorbidity” where the WL group had slightly more disorders than the SH group (**χ*^2^* = 5.85, *p* = 0.016).*

The patients’ use of medication was recorded at the initial interview. Patients using benzodiazepines discontinued these prior to treatment. Compliance was confirmed in the post-treatment assessment. Patients had no other treatment during the four days, as confirmed by medical records.

### Assessment

Patients had a diagnostic- (First et al., 2015) and Y-BOCS interview with an independent assessor by phone one week prior to starting treatment. Patients were re-assessed by an independent assessor within one week after completing treatment and at 3-month follow-up. For further procedural details regarding the RCT, please refer to Launes et al. (2019b). Following the assessment after three months of SH or WL, patients who wanted further treatment were offered participation in a B4DT group. The assessment procedures were the same for the patients that first received SH or WL. Inter-rater reliability was checked with respect to OCD diagnosis and Y-BOCS. For the OCD diagnosis the kappa value was 1.00, while the intra-class correlation for Y-BOCS was 0.97.

#### Measures

##### OCD symptoms

The Y-BOCS (Goodman et al., 1989a, b) is a 10-item interview, and was used as the primary outcome measure in this study. Ten items are rated on a 0–4 scale, and total score ranges from 0 to 40. Psychometric properties are good (Goodman et al., 1989a, b).

Two self-report measures of OCD symptoms were also included. The Dimensional Obsessive Compulsive Scale Short-Form (DOCS-SF; Eilertsen et al., 2017) is a five item version of the original 28-item version of DOCS (Abramowitz et al., 2010). The five items are rated on a scale from 0 to 8 and concern time spent, avoidance, distress, interference with functioning, and difficulty disregarding obsessions and refraining from compulsions. The total score ranges from 0 to 40.

The second self-report measure of OCD symptoms was the Obsessive-Compulsive Inventory-Revised (OCI-R; Foa et al., 2002), an 18-item scale. Items are rated on a 0–4 scale, and total score range from 0 to 72. The OCI-R has good psychometric properties (Foa et al., 2002; Solem et al., 2010).

In adition to measures of OCD-symptoms, the study included measures of comorbid symptoms of depression and generalized anxiety. The Patient Health Questionnaire-9 (PHQ-9; Kroenke et al., 2010) is a 9-item scale assessing symptoms of depression. Items are rated on a 0–3 scale, and total score ranges from 0 to 27. The PHQ-9 has sound psychometric properties (Kroenke et al., 2010).

The Generalized Anxiety Disorder Scale (GAD-7; Spitzer et al., 2006) is a 7-item scale assessing symptoms of generalized anxiety. Items are rated on a 0–3 scale, and total score ranges from 0 to 21. Psychometric properties of the GAD-7 are good (Spitzer et al., 2006; Beard and Bjorgvinsson, 2014; Rutter and Brown, 2017).

In addition to the symptom scales, we also included a measure of satisfaction with treatment. The Client Satisfaction Questionnaire 8 (CSQ-8; Nguyen et al., 1983) is an 8-item scale assessing satisfaction with treatment received. Items are rated on a 0–4 scale, and total score ranges from 8 to 32. Psychometric properties of the CSQ-8 are good (Larsen et al., 1979; Nguyen et al., 1983).

### Therapists

There were equal number of patients and therapists in each group, and each group was led by an expert on the treatment format. All therapists were trained in delivering the treatment (Kvale and Hansen, 2014). Prior to acting as a B4DT therapist, each had been evaluated as competent by two independent assessors in a minimum of two B4DT groups. Specific competencies in conducting the LET-intervention (see below) were evaluated by a B4DT expert watching videotaped sessions. The leaders of the groups had participated as therapists in at least six B4DT groups in order to be certified as competent. Then they acted as leaders of a minimum of two B4DT groups prior to the trial. Therapists in the present study included five psychologists, two psychiatric nurses, and two psychiatrists.

### The B4DT Format

A detailed description of the treatment format is given in Launes et al. (2019b). Prior to the treatment, the patients were provided with oral and written information about the content of the four days.

#### The LET-Technique

Prior to exposure treatment, patients are taught to pay close attention to the way the exposures are done, and one of the main features of the 4-day treatment, namely the “LEan into The anxiety” (LET-technique) is demonstrated. Patients have to approach stimuli that elicit anxiety/discomfort and act in a manner that is incompatible with their OCD. During the two days of exposure treatment, the patients are assisted when they practice the LET-technique, and encouraged and assisted to employ the approach in all situations that elicit anxiety/discomfort.

#### Day 1, Psychoeducation and Treatment Preparation (3–4 h)

Participants receive psychoeducation in which they are provided with explanations for the treatment and for the techniques that are seen as essential. The psychoeducation is manualized using a PowerPoint presentation with a text-file detailing the content to be presented. The patients share their suggested exposure tasks with the group, and individual plans are made together with input from the group leader.

#### Day 2 and 3, (8 h + 1.5 h Psychoeducation for Family)

Throughout Day 2 and 3, patients practice individually tailored and therapist assisted ERP. In the evening patients are to continue with self-administrated training. Exposure sessions are interspaced with brief group meetings where each patient share their ratings of their own exposure performance with the group. They evaluate their own LET-performance on a 1–6 scale. A score of 6 indicates that they were “leaning fully into” the anxiety. Scores below 6 are explored by the group leader, and the patients are given advice and encouragement aimed at giving them courage to “not hold back” in the next exposure. Patients were given the opportunity to invite relatives and friends to a psychoeducation session in the afternoon of day 3.

#### Day 4, Summarizing and Relapse Prevention

Day 4 is used for summarizing and planning self-exposures for the next three weeks. Patients are taught to be their own therapists. They make their own exposure plans to be performed the first three weeks after the treatment. Written information concerning procedures and upcoming assessments is handed out, and the group ends with the patients providing feedback on their experiences with treatment.

#### Homework Assignments and Follow-Up Visit

Patients log homework (exposures) completed for a 3-week period after treatment. About 12 weeks after treatment, patients meet for an individual follow-up session at the clinic. This session is intended to help the patient summarize their experiences after completing treatment, and if necessary, to refresh the treatment principles. This follow-up session did not involve exposure exercises.

### Classification of Treatment Response and Remission

An adaptation of the international consensus criteria was used for assessing treatment response (Mataix-Cols et al., 2016), not including the criterion for change in Clinical Global Impression. A treatment response involves a ≥35% reduction in Y-BOCS score. Remission is defined as meeting the treatment response criterion as well as having a post-treatment or follow-up Y-BOCS score of 12 or less.

### Statistical Analysis

There were few instances of missing data. For Y-BOCS and the self-report scales there were four patients missing data at post-treatment-2. However, all four patients attended the follow-up assessment, and their scores from follow-up were therefore carried backward to replace their missing post-treatment-2 score. Imputation using “next observation carried backward” is one of the imputation methods giving least bias in scores (Mundahl Engels and Diehr, 2003). There was only one patient missing self-report data at follow-up. This patient was given his/her post-treatment score as follow-up score (last observation carried forward). One patient had missing data on the CSQ-8, which was not replaced.

Paired sample *t*-tests were first used to investigate if the SH or WL groups had any significant improvement on Y-BOCS scores (post-treatment-1). Paired sample *t*-tests were also used to investigate whether the two groups had a significant improvement after being given the B4DT at post-treatment-2 and 3-month follow-up. Independent sample’s *t*-tests were used to compare the two groups at post-treatment-2 and follow-up. Possible differences in change scores (from pre-treatment to post-treatment-2 and follow-up) between participants given immediate B4DT and delayed B4DT were also compared using independent samples *t*-tests.

Treatment outcome was further analyzed using repeated measures ANOVA with three different times of assessment (pre-treatment, post-treatment, and follow-up). This analysis was used for all outcome measures (Y-BOCS, PHQ-9, GAD-7, OCI-R, and DOCS-SF).

## Results

A summary of the Y-BOCS scores for the SH- and WL-conditions is displayed in Table 2. Paired sample *t*-tests showed that there was no significant effect of the WL at post-treatment-1, while there was a significant but small improvement observed for the SH-condition. The patients then received the B4DT after the post-treatment-1 assessment. Irrespective of which original condition the patients belonged to they achieved a very large and significant improvement at the post-treatment-2 assessment. The change scores (pre-treatment to post-treatment 2/follow-up), was not significantly different between the conditions at post-treatment 2; *t*(24) = 0.164, *p* = 0.871, or at follow-up; *t*(24) = 0.35, *p* = 0.73. Participants from the two groups were therefore merged together into one group for further analyses.

**TABLE 2:** Y-BOCS means (SDs) for the two groups at the four assessment points.

	**Pre**	**Post-1**	**Post-2**	**3m F-U**	**Pre–Post-1**		**Pre–Post-2**	
			
	**Y-BOCS M (SD)**				***t***	***p***	***t***	***p***
Immediate B4DT (*n* = 16)	26.75 (4.23)		10.90 (4.35)	8.56 (5.75)				
WL (*n* = 13)	27.08 (3.59)	26.77 (4.04)	10.62 (2.22)	10.00 (5.85)	0.39	0.71	12.87	<0.0001
SH (*n* = 13)	29.15 (3.46)	26.85 (3.26)	12.99 (3.59)	11.08 (6.09)	2.93	0.01	12.62	<0.0001

*Y-BOCS, Yale-Brown Obsessive Compulsive Scale; B4DT, Bergen 4-day treatment; WL, waiting list; SH, self-help; Pre, pre-treatment; Post-1, post-treatment after WL or SH; Post-2, post-treatment after B4DT; 3m F-U, 3-month follow-up after B4DT.*

Change in Y-BOCS scores after being given the B4DT was compared between patients given immediate B4DT (*N* = 16) and patients originally in the SH or WL condition (*N* = 26). The immediate B4DT group had a mean change score of 15.85 (5.85) at post-treatment compared to 16.31 (4.52) for the SH/WL patients, *t*(40) = 0.287, *p* = 0.775. There were also a non-significant difference at follow-up, as immediate B4DT showed a mean change score of 18.19 (5.95) compared to 17.58 (7.18) for the WL/SH group, *t*(40) = 0.285, *p* = 0.777. Thus, for the analysis of clinical change all patients were combined into one group having received the B4DT, either directly or after another condition.

### Clinically Significant Change in OCD-Severity

The clinical improvements at post-treatment and follow-up are presented in Table 3. Of the 42 patients given the B4DT, 25 (59.5%) were remitted at post-treatment, 13 (31.0%) had a treatment response, and 4 (9.5%) showed no change. At 3-month follow-up 30 patients (71.4%) were remitted, 8 (19.0%) had a treatment response, and 4 (9.5%) showed no change. The 25 patients classified as remitted at post-treatment had a stable improvement as 24 (96%) were still remitted at follow-up, while one patient was now classified as a treatment responder. In other words, there were no clear signs of relapse. There were 13 patients classified as having treatment response at post-treatment, and of these five (38%) achieved remission at follow-up, five remained responders, while three showed no change at follow-up. Four patients were classified as having no change at post-treatment, and of these one patient achieved remission at follow-up, two were classified as treatment responders, and one was still classified as having no change. No patients deteriorated.

**TABLE 3:** Clinical improvement rates for B4DT atpost-treatment and follow-up.

		**3-month follow-up**				**Total**
		**Remission**	**Response**	**No change**	**Deterioration**	
Post-treatment	Remission	24	1	0	0	25
	Response	5	5	3	0	13
	No change	1	2	1	0	4
	Deterioration	0	0	0	0	0
	Total	30	8	4	0	42

*Treatment response was based on a modification of the international consensus criteria (Mataix-Cols et al., 2016). Response: ≥35% reduction in Y-BOCS score. Remission: meeting the response criterion and a post-treatment/follow-up score of 12 points or less.*

### Secondary Outcome Measures

Table 4 displays the results for Y-BOCS and secondary outcome measures at pre-treatment, post-treatment, and 3-month follow-up. Repeated measures ANOVA found a significant effect of time for all five measures. The effect size was highest for change in OCD measures and lowest (but moderate) for change in depression.

**TABLE 4:** Means (SDs) and effect sizes for the outcome measures at the various assessment points.

	**Pre**	**Post**	**FU**	***F***	***p***	**Partial η*^2^***	***d* pre-post**	***d* pre-FU**
Y-BOCS	27.08 (3.32)	11.80 (3.16)	10.54 (5.87)	136.98	<0.001	0.85	4.60	4.98
DOCS-SF	25.85 (4.49)	17.15 (6.09)	15.00 (9.13)	30.02	<0.001	0.55	1.94	2.42
OCI-R	25.19 (12.88)	16.73 (12.51)	14.85 (13.53)	18.12	<0.001	0.42	0.66	0.80
GAD-7	13.24 (4.32)	9.38 (3.95)	8.12 (4.46)	15.91	<0.001	0.39	0.89	1.19
PHQ-9	12.81 (4.36)	9.88 (3.65)	9.73 (4.90)	6.23	0.004	0.20	0.67	0.71

*Test of within subjects, *N* = 26. Cohen’s *d* = *M*_*pre*_–*M*_*post*_/*SD*_*pre*_. Mauchly’s test of sphericity was significant for OCI-R, but not for the other measures. Y-BOCS, Yale-Brown Obsessive Compulsive Scale; DOCS-SF, Dimensional Obsessive Compulsive Scale Short-Form; OCI-R, Obsessive-Compulsive Inventory-Revised; GAD-7, Generalized Anxiety Disorder Scale; PHQ-9, Patient Health Questionnaire-9.*

### Additional Analyses

Patients using psychotropic medication did not differ on OCD-severity at pre-treatment, *t*(24) = 0.33, *p* = 0.74, from those not taking such medication. However, they showed tendencies toward having higher Y-BOCS scores at post-treatment (*M* = 13.2, SD = 4.0 vs. *M* = 10.8, SD = 1.9) and follow-up (*M* = 13.0, SD = 6.6 vs. *M* = 8.7, SD = 4.8), *p* = 0.048 and *p* = 0.066, respectively. Similar observations were made for the DOCS-SF data, but not for the other outcome measures (PHQ-9, GAD-7, and OCI-R). There were no significant differences in client satisfaction scores, *t*(22) = 0.09, *p* = 0.93.

### Treatment Satisfaction

Data on treatment satisfaction is presented in Table 5. The majority (96%) were happy with the quality of service, and all participants would have recommended it to a friend with a similar condition. All participants also rated the overall satisfaction with the treatment as positive. The mean satisfaction score of 28.96 (SD = 3.06) was comparable to patients in the immediate B4DT condition who scored 29.69 (SD = 2.62), a non-significant difference.

**TABLE 5:** Patients’ treatment satisfaction with B4DT after initial self-help or waiting list.

**Item**	**Satisfaction rating**			
	**1**	**2**	**3**	**4**
Quality of service	0	1	10	14
Kind of service	0	0	11	14
Met needs	1	0	15	9
Recommend to friend	0	0	2	23
Amount of help	0	0	10	15
Dealt with problems	0	1	6	18
Overall satisfaction	0	0	11	14
Come back	0	2	3	20

**N* = 25. Items are scored on a scale from 1 (low satisfaction) to 4 (high satisfaction).*

## Discussion

The present study was a follow-up of a recently conducted RCT for OCD where the experimental group was treated with the newly developed B4DT and the two control conditions consisted of patients that either were allocated to SH or WL. The results from the controlled trial (Launes et al., 2019b) showed that compared to SH- and WL-participants the patients in the B4DT reported significantly lower scores on OCD severity, depression, generalized anxiety, as well as higher social and work-related functioning, at post-treatment. The patients who were in need of further treatment after SH or WL were offered B4DT, and the aims of the present study were to investigate if the clinical changes after B4DT are also seen in patients with prior experience of SH or a WL, and if patients who had received an unsuccessful SH treatment improved as much as patients who had received the B4DT as the initial intervention.

The results indicate that both the patients who had previously received SH and those in the WL condition benefited significantly from the B4DT, and that they did not differ in treatment effect at 3-month follow-up from the patients who had received the B4DT immediately. At post-treatment and follow-up 90.5% showed a treatment response, and the remission rates were 58% at post-treatment and 73.1% at follow-up. This corresponds well with previous research on the B4DT (e.g., Kvale et al., 2018). Overall, treatment gains made from pre- to post-assessment were maintained and slightly improved at the 3-month follow-up, with no difference between the conditions in terms of reduction of OCD-, depression- and general anxiety symptoms. There were also no differences in experienced satisfaction with the B4DT even for the patients that first had been on a WL or participated in a SH program for OCD.

There are only three studies investigating the effect of stepped care treatment for OCD, and they suffer from sample size and attrition issues. In a pilot study of stepped care, 11 patients started with SH (step 1), followed by self-directed ERP with minimal therapist contact (step 2) if they did not respond to the first step of treatment, and step 3 was traditional therapist directed ERP (Tolin et al., 2005). Of the 11 who started treatment two patients responded after step 1, two responded after step 2, and two responded after step 3 (total of 6 responders, 55% of ITT). Gilliam et al. (2010) also published a small open trial of stepped care treatment for 14 patients with OCD. Participants first received self-directed ERP (step 1) followed by therapist-directed ERP (step 2). In that study, five patients responded after step 1, while only one patient responded at follow-up (total response rate of 43% for ITT). Results from step 2 was somewhat discouraging, however, it is difficult to conclude whether the first step affected treatment response in step 2. A somewhat larger and controlled study assigned 19 patients to stepped care [12 completers, seven (37% of ITT) responders at follow-up], and 15 assigned to standard ERP [10 completers, three (20% of ITT) responders at follow-up] (Tolin et al., 2011). The low treatment response rate still leaves the question as to whether stepped care is a suitable treatment approach for people with OCD. Furthermore, since this study dating back to 2011 there has, to the best of our knowledge, been no study published on stepped care for OCD. Along with the results of the current study, there is no clear evidence that stepped care treatment should be the preferred treatment of choice. However, there is also no clear evidence that stepped care hinders subsequent interventions.

The results suggest that there should be no methodological or obvious ethical concerns to first randomize OCD-patients to 3-month of WL or a SH program before they receive the B4DT. Given that patients usually have to wait before they receive treatment, this finding is interesting because it underscores that it is likely that patients can benefit from the B4DT even if they do not receive the treatment immediately. This can be of value, e.g., when patients express an urge to initiate treatment as soon as possible. Patients who had received SH did not gain more from the B4DT post-treatment or at follow-up as compared to the WL-patients. On the other hand, the patients with prior SH, were not at a disadvantage regarding the benefit they experienced following the B4DT. It is noteworthy that the SH was delivered without assistance from a therapist and that the patients were guaranteed B4DT after the SH treatment if they needed further treatment.

There are some limitations of the current study such as limited sample size and lack of long-term follow-up. However, studies using the B4DT with larger sample sizes and long-term follow-up assessments have indicated that the results are consistent. Another limitation concerns the fact that the SH condition was not assisted. It is likely that assisted SH could yield better treatment results (Andersson et al., 2012, 2015). Furthermore, it is unclear what effect the B4DT would have for patients who have been treated with assisted SH. Although the study included all consecutive referrals to the clinic, there might, however, be noted that during the referral period there was an ongoing national study that targeted specifically patients with OCD that previously had received CBT. Another limitation concerns the unbalanced gender distribution. Furthermore, which components of the 4-day format that can be seen as critical for outcome was not the focus of the current study. However, future research should consider comparing the B4DT with the same treatment delivered across 12 weeks with a single therapist and individual treatment given in the 4-day format. This could allow for inferences concerning specific components of the treatment such as the concentration of the treatment and the effects of working in groups.”

The results from this RCT provide evidence that most OCD patients treated with B4DT, delivered at another site than the originators’ clinic, achieve highly comparable outcomes at post-treatment and 3-month follow-up, as a number of open trials have reported (Havnen et al., 2014, 2017; Hansen et al., 2018; Kvale et al., 2018; Davíðsdóttir et al., 2019; Launes et al., 2019a, b). In addition, the study replicated previous findings concerning refusal and attrition rates. Replication studies on the B4DT format are needed from other sites, countries and cultures.

## Data Availability Statement

The datasets generated for this study are available on request to the corresponding author.

## Ethics Statement

The trial received ethical approval from the Regional Committee for Research Ethics (REK Vest, 2016/794) and was registered in ClinicalTrials.gov (Identifier: NCT02886780). Patients still in need of treatment after the SH intervention and the WL, were for ethical reasons granted participation in a B4DT group if they wanted. Also, the length of the SH and WL conditions were not longer than the ordinary waiting period at the clinic at the time when the trial was initiated. The patients/participants provided their written informed consent to participate in this study.

## Author Contributions

GK, L-GÖ, BH, KH, and GL contributed to the study design. GL and KH contributed to the data collection. All authors contributed to the statistical analysis, interpretation of the data, and drafting of the manuscript, and approved the final version.

## Conflict of Interest

The authors declare that the research was conducted in the absence of any commercial or financial relationships that could be construed as a potential conflict of interest.
